# Supplementary material for: Formation of Nanofoam carbon and re-emergence of Superconductivity in compressed CaC6
Source: Sci Rep. 2013 Nov 26;3:3331. doi: 10.1038/srep03331 (PMC3840379; doi:10.1038/srep03331)
Supplement: Supplementary Information — CaC6-Suppl-Info [file srep03331-s1.pdf]

Supplementary information

Formation of Nanofoam carbon and re-emergence of Superconductivity in compressed CaC_6

Yan-Ling Li,[1] Wei Luo,[2] Xiao-Jia Chen,[3,4] Zhi Zeng[4] , Hai-Qing Lin[5] & Rajeev Ahuja[2,6]

[1] School of Physics and Electronic Engineering, Jiangsu Normal University, 221116, Xuzhou, People's Republic of China

[2] Condensed Matter Theory Group, Department of Physics and Astronomy, Uppsala University, P.O. Box 516, SE-751 20 Uppsala, Sweden

[3] Geophysical Laboratory, Carnegie Institution of Washington, Washington, DC 20015, USA

[4] Key Laboratory of Materials Physics, Institute of Solid State Physics, Chinese Academy of Sciences, Hefei 230031, People's Republic of China

[5] Beijing Computational Science Research Center, Beijing 100089, People's Republic of China

[6]Applied Material Physics, Department of Materials Science and Engineering, Royal Institute of Technology (KTH), SE-100 44, Stockholm, Sweden

This file includes Three Tables and nine Figures.

Supplementary Table S1. Structures of stable phases of CaC_6 and carbon foam. Only the fractional coordinates of symmetry inequivalent atoms are given. For *C222* phase, under pressure, it is easy to transform into *Cmmm* symmetry, which is verified by total enthalpy calculation.

Pressure (GPa)	Space group (No.)	Lattice parameters $(a, b, c, \alpha, \beta, \gamma)$ (Å, ^{0})			Atomic fractional coordinates			
0	*R-3m*	5.1683	5.1683	5.1683	Ca 4*i*	0.0000	0.0000	0.0000
	(164)	49.7	49.7	49.7	C1 6*g*	0.1667	0.8333	0.5000
19	*C222*	9.1472	3.6947	3.5964	Ca 2*a*	0.0000	0.0000	0.0000
	(29)	90	90	90	C1 4*f*	0.4213	0.0000	0.5000
					C2 8*l*	0.1751	0.8082	0.4999
39	*Pmmn*	5.8904	2.4893	6.4903	Ca 2*b*	0.0000	0.5000	0.7425
	(59)	90	90	90	C1 2*b*	0.0000	0.5000	0.3159
					C2 2*a*	0.0000	0.0000	0.1766
					C3 4*f*	0.1994	0.0000	0.0286
					C4 4*f*	0.2791	0.0000	0.5537
126	*Cmcm*	2.3828	16.1113	4.0533	Ca 4*c*	0.0000	0.4874	0.2500
	(63)	90	90	90	C1 8*f*	0.0000	0.1044	0.4303
					C2 8*f*	0.0000	0.2705	0.5647
					C3 8*f*	0.0000	0.3551	0.4371
0	C_6:*Cmcm*	5.9354	6.3121	2.4675	C1 4*c*	0.0000	0.5716	0.2500
	(63)	90	90	90	C2 8*g*	0.2949	0.2163	0.2500

Supplementary Table S2. The bond lengths and bond angle of carbon nanofoam (C_6, space group *Cmcm*), *Pmmn*, *Cmcm* phases of compressed CaC_6 compared to experimental data of graphite and diamond.

Structure	Density(g/cm^3)	Bond length (Å)			Bond angle (^{0})
Graphite	2.27	sp^2-sp^2	1.421	sp^2-sp^2-sp^2	120.00
diamond	3.54	sp^3-sp^3	1.545	sp^3-sp^3-sp^3	109.47
			0 GPa		
nanofoam	2.589	sp^2-sp^2	1.410	sp^2-sp^3-sp^2	106.24
C_6 (*Cmcm*)		sp^2-sp^3	1.522	sp^3-sp^3-sp^3	107.55
		sp^3-sp^3	1.529	sp^3-sp^3-sp^2	110.77
				sp^3-sp^2-sp^2	118.93
				sp^2-sp^2-sp^2	120.11
Pmmn	3.499	sp^2-sp^2	1.470,1.513	sp^2-sp^3-sp^2	101.70,112.70
		sp^2-sp^3	1.582,1.620	sp^3-sp^3-sp^3	107.43
		sp^3-sp^3	1.596	sp^3-sp^3-sp^2	109.14,111.94
				sp^3-sp^2-sp^2	117.00,118.73
				sp^2-sp^2-sp^2	116.46,122.24
			39 GPa		
Pmmn	3.914	sp^2-sp^2	1.426,1.467	sp^2-sp^3-sp^2	101.59,113.92
		sp^2-sp^3	1.520,1.552	sp^3-sp^3-sp^3	108.02
		sp^3-sp^3	1.538	sp^3-sp^3-sp^2	108.69,111.80
				sp^3-sp^2-sp^2	117.07,119.01
				sp^2-sp^2-sp^2	116.05,121.50
			126 GPa		
Cmcm	4.788	sp^2-sp^2	1.462	sp^3-sp^2-sp^2	111.58
		sp^2-sp^3	1.461	sp^2-sp^3-sp^3	111.58
		sp^3-sp^3	1.458,1.460<br>1.502,1.517	sp^3-sp^3-sp^3	107.19, 109.41<br>110.78, 111.06
				sp^2-sp^3-sp^2	109.26
				sp^3-sp^2-sp^3	109.26

[1] Kittel, C. *Introduction to solid state physics* (8th edition, 2004).

Supplementary Table S3. The calculated values of the density of states at the Fermi level *N(0)*, phonon logarithmic average ω_{log}, electron-phonon interaction λ, and superconducting transition temperature T_c of CaC_6 for the *Pmmn* structure at different pressures. μ^* is the Coulomb pseudopotential parameter.

**Space group**	***P*** **(GPa)**	***N(0)*** **(/Ry)**	ω_{log} **(*K*)**	λ	T_c (μ^*=0.11)	T_c (μ^*=0.115)	T_c (μ^*=0.12)
Pmmn	39	15.645	642	0.445	3.87	3.48	3.12
	51	15.58	663	0.587	12.7	11.9	11.2
	78	15.378	623	0.638	15.4	14.7	13.9
	117	14.830	728	0.461	5.23	4.74	4.28

Figure captions

Supplementary Fig. S1. Projected density of states of *Pmmn* structure at phase transition pressure. One can see that there is strong hybridization between Ca-*d* electrons and *p* electrons of C3 and C4 (forming sp^2 hybridized zigzag chains along y-axis direction). sp^3 hybridized C1 and C2 have very little contribution to Fermi level because of their saturated chemical bonds (see also Fig. S3).

Supplementary Fig. S2. Projected density of states of *Cmcm* structure at phase transition pressure. Weak hybridization between Ca-*d* electrons and C-*p* electrons is observed at Fermi level. Pressure brings about *d* energy level move to valence bands, which does good to decrease the total energy of system so as to compensate pressure-induced energetic increase, making it be favorable one energetically.

Supplementary Fig. S3. Electronic local functional (ELF) and structural schematic diagram for *Pmmn* and *Cmcm* phases at phase transitional pressure. Electronic local functional (ELF)[2] for *Pmmn* structure (a), nanofoam schematic diagram constructed by carbon atoms in *Pmmn* phase, centered with Ca ion chains (b) (unit: Å), Cmcm structure along (100) direction (c), and ELF for *Cmcm* structure (d) and (e). In (b) and (c), inequivalent carbon atoms depicted using different color balls, and calcium atoms shown using green balls. For *Pmmn* structure at 39 GPa, sp^2 were connected by sp^3 with C-C chemical bonding (length is 1.552 or 1.520 Å). Along y-axis direction, for sp^2 carbons, there are two types of C-C bonding with different lengths in zigzag chain (1.467 and 1.426 Å). While for sp^3 carbons, the bonding length in zigzag chains along y direction is 1.538 Å. For *Cmcm* structure at 126 GPa, the bonding length could be seen in Table S1.

Supplementary Fig. S4. Phonon dispersion of *Pmmn* phase at zero pressure obtained using Phonopy code. Obviously, it is stable dynamically.

Supplementary Fig. S5. Phonon dispersion of *Cmcm* carbon nanoporous (appeared in phase III) at zero pressure obtained using Phonopy code[3].

Supplementary Fig. S6. At zero GPa, band structure of carbon foam (space group *Cmcm*) obtained by removing Ca atoms of phase III (space group, *Pmmn*). The band gap is about 0.49 eV.

Supplementary Fig S7. Four types of nanoform structures of intercalation compounds under compression. LiC_6(a), CaC_6 (b), and KC_8 (c and d).

Supplementary Fig S8. Total density of states (TDOS) for R-3m at ambient pressure, C222, Pmmn, and Cmcm phases at phase transition pressure. Obviously, pressure leads to more expanded electronic distribution compared to that of *R-3m*, which leads to lower TDOS in *C222* and *Cmcm*. However, *Pmmn* phase has comparative TDOS with that of *R-3m* phase.

Supplementary Fig S9. Phonon spectrum of three high pressure phases for CaC_6 at cold pressure calculated using Phonopy code. There are soft modes in peculiar direction for *Pmmn* and *Cmcm* phases.

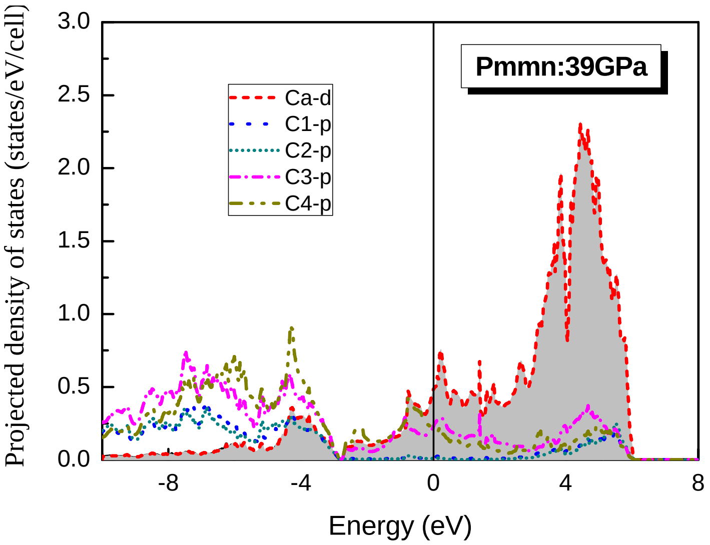

Supplementary Fig. S1. Projected density of states of Pmmn structure at phase transition pressure. One can see that there is strong hybridization between Ca-*d* electrons and *p* electrons of C3 and C4 (forming sp^2 hybridized zigzag chains along y-axis direction). sp^3 hybridized C1 and C2 have very little contribution to Fermi level because of their saturated chemical bonds (see also Fig. S3).

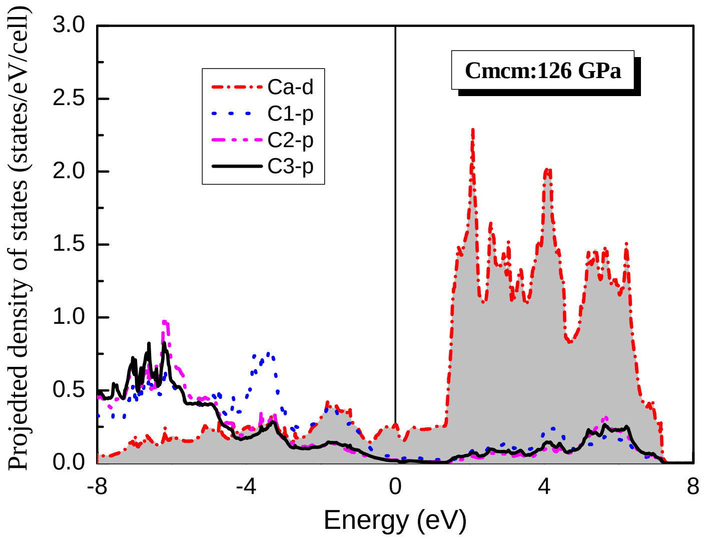

Supplementary Fig. S2. Projected density of states of *Cmcm* structure at phase transition pressure. Weak hybridization between Ca-*d* electrons and C-*p* electrons observed at Fermi level. Pressure brings about *d* energy level move to valence bands, which does good to decrease the total energy of system so as to compensate pressure-induced energetic increase, making it be favorable one energetically.

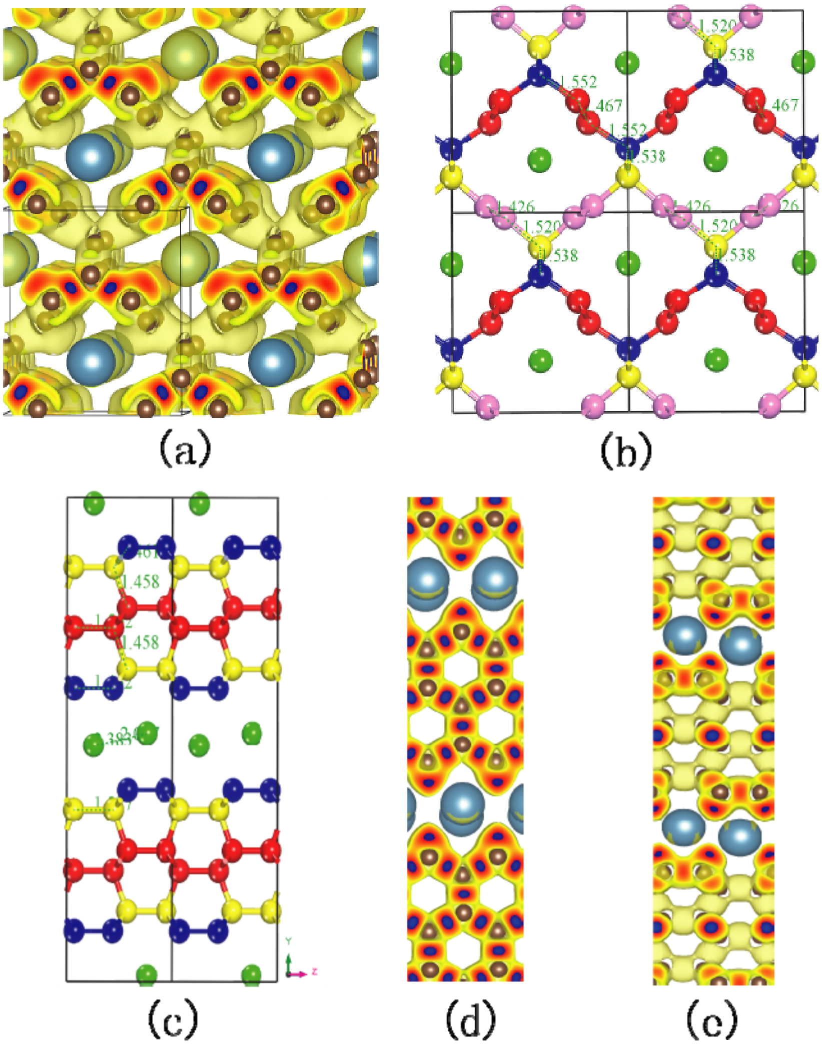

Supplementary Fig.S3. Electronic local functional (ELF)[2] for *Pmmn* structure (a), nanofoam schematic diagram constructed by carbon atoms in *Pmmn* phase, centered with Ca ion chains (b) (unit: Å), Cmcm structure along (100) direction (c), and ELF for *Cmcm* structure (d) and (e). In (b) and (c), inequivalent carbon atoms depicted using different color balls, and calcium atoms shown using green balls. For *Pmmn* structure at 39 GPa, sp^2 were connected by sp^3 with C-C chemical bonding (length is 1.552 or 1.520 Å). Along y-axis direction, for sp^2 carbons, there are two types of C-C bonding with different lengths in zigzag chain (1.467 and 1.426 Å). While for sp^3 carbons, the bonding length in zigzag chains along y direction is 1.538 Å. For *Cmcm* structure at 126 GPa, the bonding length could be seen in Table S1.

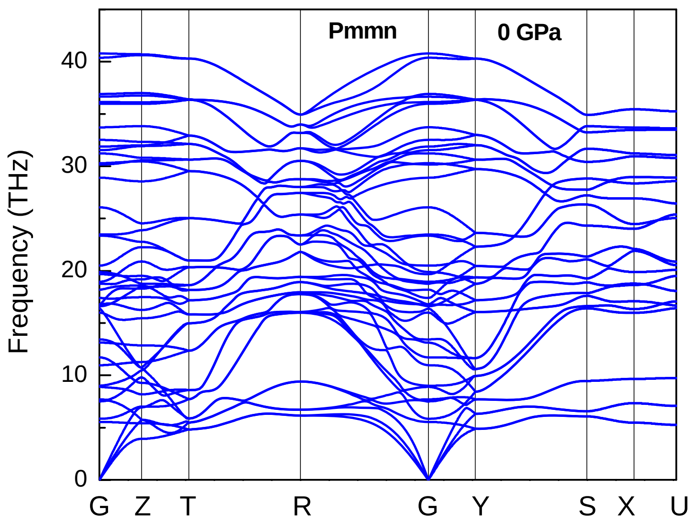

Supplementary Fig S4. Phonon dispersion of Pmmn phase at zero pressure obtained using Phonopy code. Obviously, it is stable dynamically.

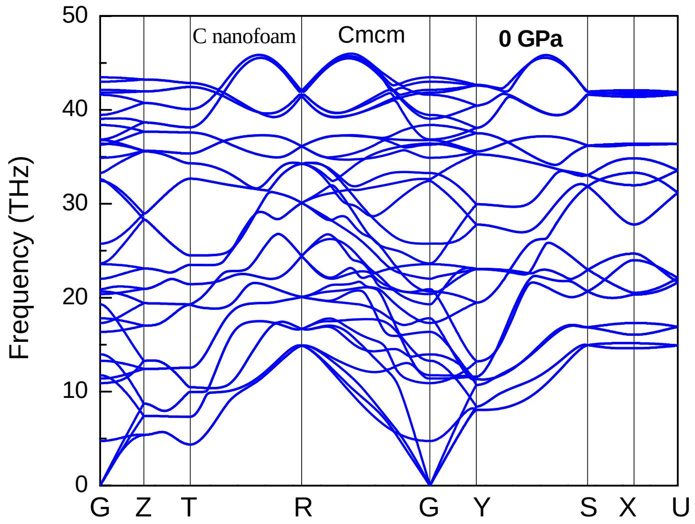

Supplementary Fig. S5. Phonon dispersion of Cmcm carbon nanoporous at zero pressure obtained using Phonopy code[3].

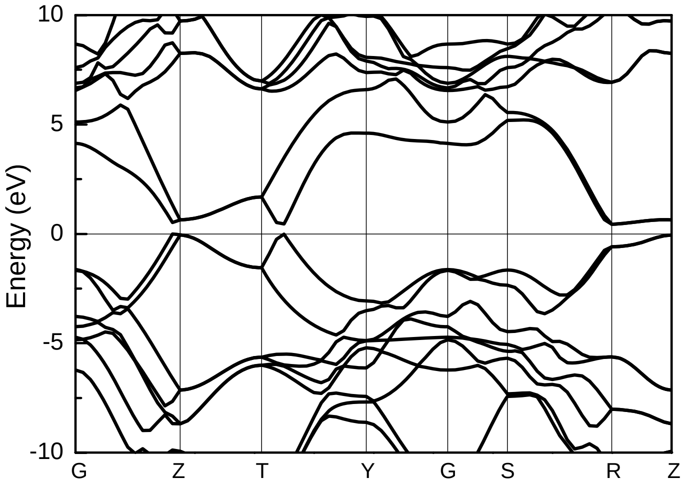

Supplementary Fig. S6. At zero GPa, band structure of carbon foam (space group *Cmcm*) obtained by removing Ca atoms of phase III (space group, *Pmmn*). The band gap is about 0.49 eV.

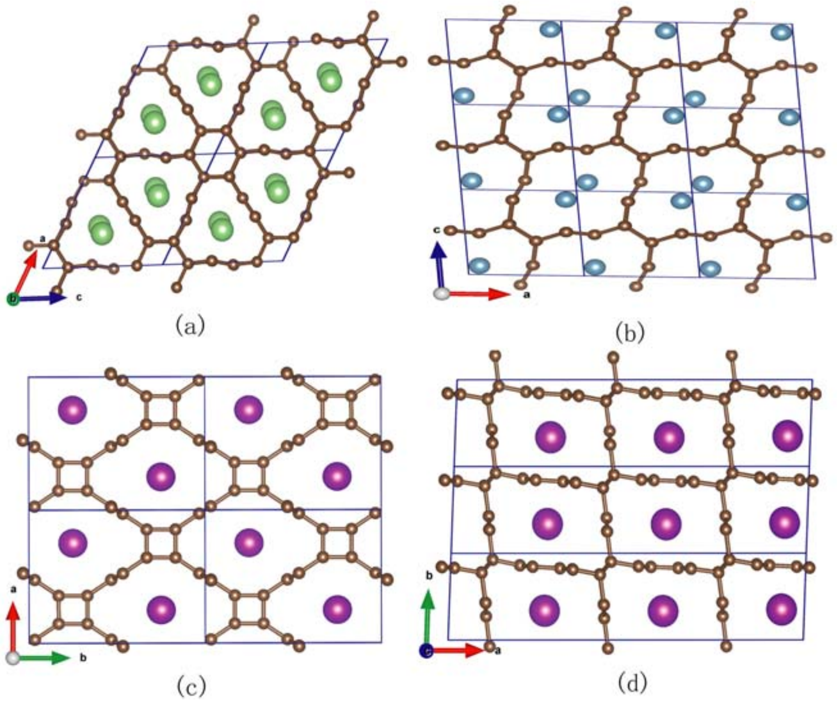

Supplementary Fig. S7. Four types of nanoform structures of intercalation compounds under compression. LiC_6(a), CaC_6 (b), and KC_8 (c and d).

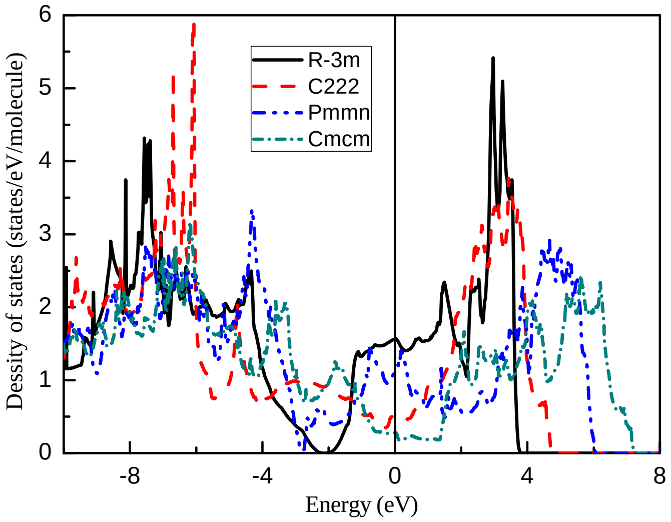

Supplementary Fig S8. Total density of states (TDOS) for *R-3m* at ambient pressure, *C222*, *Pmmn*, and *Cmcm* phases at phase transition pressure. Obviously, pressure leads to more expanded electronic distribution compared to that of *R-3m*, which leads to lower TDOS in *C222* and *Cmcm*. However, *Pmmn* phase has comparative TDOS with that of *R-3m* phase.

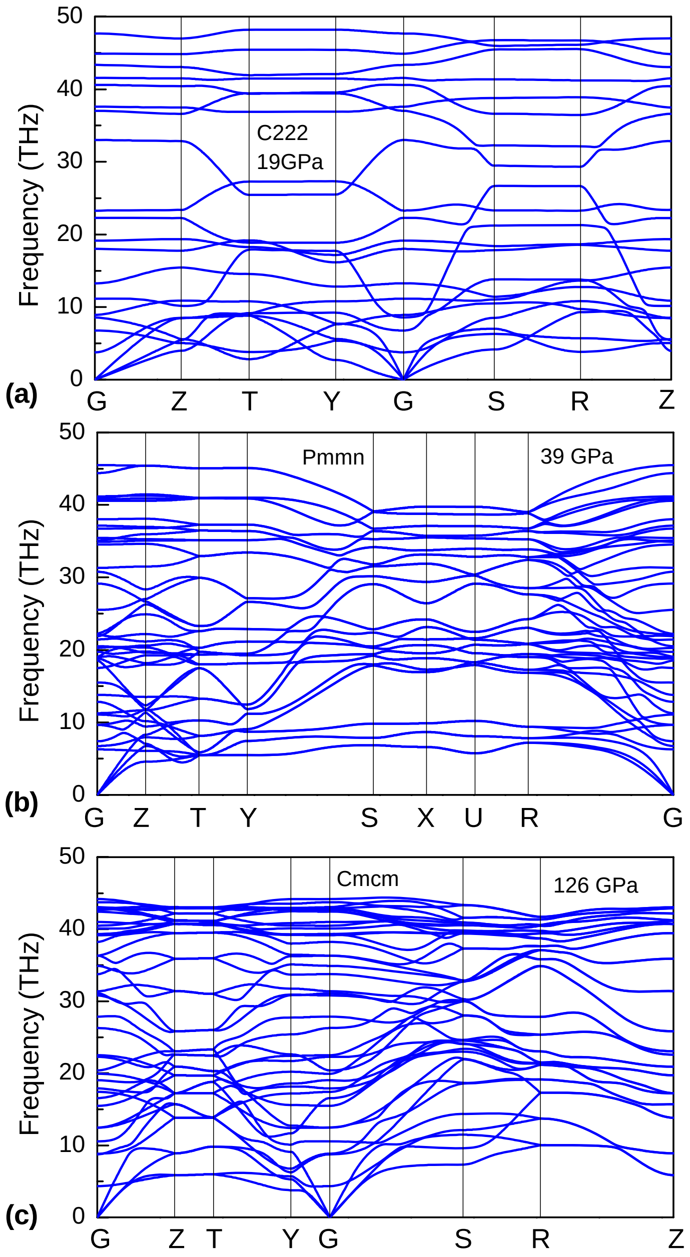

Supplementary Fig. S9. Phonon spectrum of three high pressure phases for CaC_6 at cold pressure calculated using Phonopy code. There are soft modes in peculiar direction for *Pmmn* and *Cmcm* phases.

Reference:

1. Kittel C. *Introduction to solid state physics* (8th edition, 2004).
2. Momma K, and Izumi F (2011) VESTA 3 for three-dimensional visualization of crystal, volumetric and morpholopy data. *J. Appl. Crystallogr.* **44**, 1272-1276.
3. Togo A, Obe F, and Tanaka I (2008) First-principles calculations of the ferroelastic transition between rutile-type and $CaCl_2$-typre SiO_2 at high pressures. *Phys. Rev. B* **78**, 134106.
